# Occupational benefit perception of acute and critical care nurses: A qualitative meta-synthesis

**DOI:** 10.3389/fpubh.2022.976146

**Published:** 2022-09-30

**Authors:** Shuyang Liu, Xia Duan, Peng Han, Haiyan Shao, Jinxia Jiang, Li Zeng

**Affiliations:** ^1^Emergency Department, Shanghai Tenth People's Hospital, School of Medicine, Tongji University, Shanghai, China; ^2^Nursing Department, Shanghai First Maternity and Infant Hospital, School of Medicine, Tongji University, Shanghai, China; ^3^Department of Nursing, Tongji Hospital, School of Medicine, Tongji University, Shanghai, China

**Keywords:** acute and critical care, nurses, qualitative review, meta-synthesis, occupational benefit perception

## Abstract

**Background:**

With the development of society, nurses have an increasingly more important role in the medical team. At the same time, due to various reasons, the number of active nurses is continuously decreasing, and the shortage of nursing personnel is becoming ever more serious. The COVID-19 pandemic made these clinical problems more serious. As the department with the greatest work pressure and the most intense pace, acute and critical care nurses are already facing serious problems related to job burnout and dismission. In the context of the COVID-19 pandemic, these problems should be solved urgently. Furthermore, with the rise of positive psychology, many scholars are turning their research direction to the positive professional experience of nurses so as to get inspiration to encourage nurses to face work with an optimistic attitude and guide nursing managers to better retain nursing talents.

**Objective:**

The purpose of this paper is to summarize and evaluate the positive emotional experience and professional benefit of acute and critical care specialist nurses in the process of work. So as to better interpret their occupational benefit perception and guide nursing managers in adopting positive measures and promoting the development of high-quality nursing.

**Methods:**

Cinahl plus, Embase, Medline and other twelve databases were searched for relevant literature. Meta-aggregation was used to synthesize the findings of the included studies.

**Results:**

From a total of 12 articles included in this study, 55 main results were presented, 8 new categories were integrated, and three themes were formed: professional identity, social support, and personal growth. The professional identity included: being proud of professional ability and increasing professional value; social support included: friends and family support, organizational, environmental support, peer support, and support of patients and their families; personal growth included realizing self-worth and promoting self-development.

**Conclusion:**

Hospital managers should pay attention to the positive emotional experience of nurses in work and based on this, provide practical and beneficial protection for nurses from the aspects of salary, learning opportunities, working environment, social support and internal personality, stimulate work enthusiasm, guide nurses to correctly face negative emotions and occupational pressure, and improve the sense of professional benefit.

## Introduction

With the development of society and the great advancements in medical settings, nurses represent the central and largest sector of the healthcare workforce (1). They have an important role in daily medical work but are also expected to bear the weight of unexpected medical emergencies such as the outbreak of COVID-19. Undoubtedly, nurses are patients' closest associates, commonly assuming the role of their “best friends.” However, due to high work pressure, uncertain career prospects, poor salary, response fatigue to public health events, and other reasons, the high dimission rate of nursing staff has become increasingly serious (2). Following the rapid development of medical technology and the progress of the nursing process, the shortage of nurses in this context deserves more attention and the adoption of some positive measures (3). As this issue has become a global problem, some scholars argue that solving the shortage of nurses might be the basis for advancing medical cause (4). Each country has its criteria for evaluating nurse shortages. According to previous studies, American states have nearly 700 nurses for every 10,000 people, while some African countries, such as Uganda, have a nurse-to-population ratio of approximately 6:10,000. Both these countries report nurse shortages (5). In Europe, forty-three percent of nurses plan to leave their occupation within 5 years, while in China, this phenomenon is particularly serious (6). Some scholars have studied the status quo of turnover intention and turnover rate of nurses in China, reporting that the turnover intention of nurses, which is affected by multiple factors, such as age, region, and culture, is generally at a high level (7). All of these questions affect the service quality and the rehabilitation of patients, as well as the development of medical causes and the improvement of medical levels. A shortage of nurses is expected to continue until 2030, making it imperative to take active measures to deal with nurse turnover (1).

The COVID-19 pandemic broke out in Wuhan, China, in 2019 and has spread to all parts of the world. Due to the high variability and rapid spread of the virus, there is currently a lack of specific drugs, so the preventive and control measures still mainly involve preventing respiratory transmission and vaccination. In medical staff, who have undoubtedly been on the front line, facing the danger of infection and isolation, the psychological pressure is self-evident. Some hospitals in the preview triage department reassigned paramedics to screen patients as soon as possible, preventing the epidemic from spreading in the hospital, thus reducing the staff within the department and consequently increasing workload. Currently, the normalization of the epidemic is the main response measure. Under the threat of multiple pressures, medical staff inevitably accumulates negative emotions, which in turn enforces their resignation intention. Previous studies have pointed out that COVID-19 has increased the physical, psychological, and social pressure on the medical staff, and such a high-pressure working environment has also increased the turnover of nurses. During the COVID-19 outbreak, nurses, as the main force and front-line personnel, must have close contact with infected people, and each nurse may need to wear 3–4 layers of protective equipment, including clothing, masks, goggles, gloves, and shoe covers. Wearing protective clothing for a long time may lead to hypoxia, shortness of breath, and some other adverse reactions e.g., masks may cause facial pressure injuries, while gloves affect the sense of touch and operation of nurses. Nurses may also lack the support and comfort of family members and relatives due to isolation, resulting in great psychological and mental pressure. Some patients do not have adequate knowledge of their diseases or do not fully understand medical staff, which further increases the difficulty of nurses' work. In addition, the mobility of patients in acute and critical care departments is large, and nurses come into contact with many patients, which increases their risk of infection, putting them under greater physical and mental pressure (8). COVID-19 is not only an epidemiological problem but also a socio-economic and structural problem (9).

At the forefront of the hospital, acute and critical care nurses are always confronted with urgent and unknown situations and often come into contact with stressful events such as death and serious injury of patients. As the emergency department is also more likely to be the site of violence and conflict, the working pressure of nurses is particularly prominent compared with nurses in other departments (10). At present, a series of problems caused by the shortage of nurses make the working pressure on acute critical care nurses even more challenging: long waiting times, crowded spaces, and delayed treatment make patients and their families feel anxious and irritable, which may result in aggressive behavior (11). They may need to make critical nursing decisions independently in situations where adequate information is lacking. Also, an unknown number of critically ill patients may be admitted at any given time. Furthermore, they may suffer from limited peer and social support and rest periods. Studies have suggested that acute and critical care nurses need to maintain constant vigilance and alertness (12) in order to respond to emergencies well. Long-term work pressure beyond the acceptable threshold can cause a series of psychological stress responses and job burnout; heavy workloads and hospital scheduling can lead to occupational diseases. Previous studies have shown that the dismission of nurses in acute and critical care departments is particularly serious (13), and the impact of the current epidemic has further exacerbated the nurse shortage. While the mass dismission of nurses further intensives the existing conflicts, it also causes a series of problems: the decline of medical quality, the surge of patient dissatisfaction, the regression of first-aid efficiency, and the increase of adverse clinical outcomes for patients. In addition, due to the particularity of the working environment in the acute and critical care department, nurses are required to remain calm in emergency situations and quickly and skillfully provide treatment and guidance for patients, all of which highlight the importance of work experience of nurses. However, the increasing turnover rate of nurses has led to a shortage of senior nurses, posing another problem as it takes a long time and energy to re-train new nurses (14).

Since the rise of positive psychology, more and more scholars have turned to the positive emotional experience of nurses in the professional process in order to retain nursing talents by improving nurses' professional emotions (6). Studies have pointed out that the sense of benefit and satisfaction with the nursing profession is closely related to their intention to stay on the job. According to a previous study, nurses with low levels of job satisfaction were 65 % more likely to leave than nurses with high job satisfaction (15). Therefore, the professional benefit among nurses has become a focus of attention. An in-depth understanding of nursing staff's positive emotions and job demands can provide managers with ideas for formulating more effective measures to reduce stress and the dismission rate, improve resilience and enhance professional identity, strengthen the willingness to stay on the job, awaken the enthusiasm for work, provide high-quality care to patients and ultimately lead to better clinical outcomes for patients.

In the past, a large number of studies often started from the negative factors affecting nurses' intention to stay, or used the perceived occupational benefit scale to carry out quantitative research. This paper focuses on the integration of qualitative research from the perspective of positive psychology to explore the real needs of emergency and critical care specialist nurses to make the conclusions more in-depth and applicable. This paper is aim to effectively guide nursing managers to formulate and take corresponding measures, pay attention to and meet the psychological and professional needs of nurses, retain more nursing talents, reduce the turnover rate, and promote the development of high-quality nursing.

## Methods

### Design

This is a systematic evaluation and meta-synthesis of qualitative research. Qualitative research is rooted in an interpretive paradigm of cognitive multiple socially constructed realities and aims to explore how people interact with and interpret the world (6). Qualitative research is gaining an increasingly important role in the medical field as it can often explore participants' emotional experiences more deeply than other research methods (16).

The Preferred Reporting Items for Systematic Reviews and Meta-Analysis (PRISMA) (17) was used as a basis for reporting the review. Inspired by Sandelowski et al. (18), meta-synthesis of qualitative research is based on the premise of understanding its philosophical thoughts and methodology, repeatedly reading the included literature and extracting the themes and hidden meanings so as to conduct inductive analysis, form new categories, and finally integrate new results. By synthesizing new results, a more profound and substantial explanation can be given to specific phenomena, providing a more influential and persuasive final conclusion.

### Search methods

Cinahl plus, Embase, Medline, Cochrane, Elsvier, Ovid, Pubmed, Web of Science, CNKI, VIP, Chinainfo, and Chinese Biomedical Literature databases were searched for relevant literature. Search terms “nurs^*^,” “professional benefit,” “professional benefit perception,” “sense of career benefit,” “Job satisfaction,” “retention will,” “professional identity” were included and combined using Boolean operators. These search terms were also adjusted and matched to ensure a comprehensive range of searches. The retrieval period was from database construction to April 2022.

### Eligibility criteria and study selection

#### Study design(S)

The qualitative research and qualitative part of mixed method research is included in this study. The methodologies of qualitative research is not limited, including ethnography, grounded theory, phenomenological methods, etc.

#### Participant(P)

Nurses now working in the emergency and critical care department.

#### Interest of phenomenal(I)

Positive experience and professional benefit of emergency and critical care specialist nurses in their work.

#### Context(Co)

Positive experiences of nurses who are currently working in the emergency and critical care department and will continue to work in the future.

### Exclusion criteria

The exclusion criteria are as follows: duplicate and unavailable full-text literature; non-Chinese and non-English literature; the subjects were other clinical medical workers; conference papers; case studies; official reports; book reviews; review articles and editorials.

**Figure 1:**
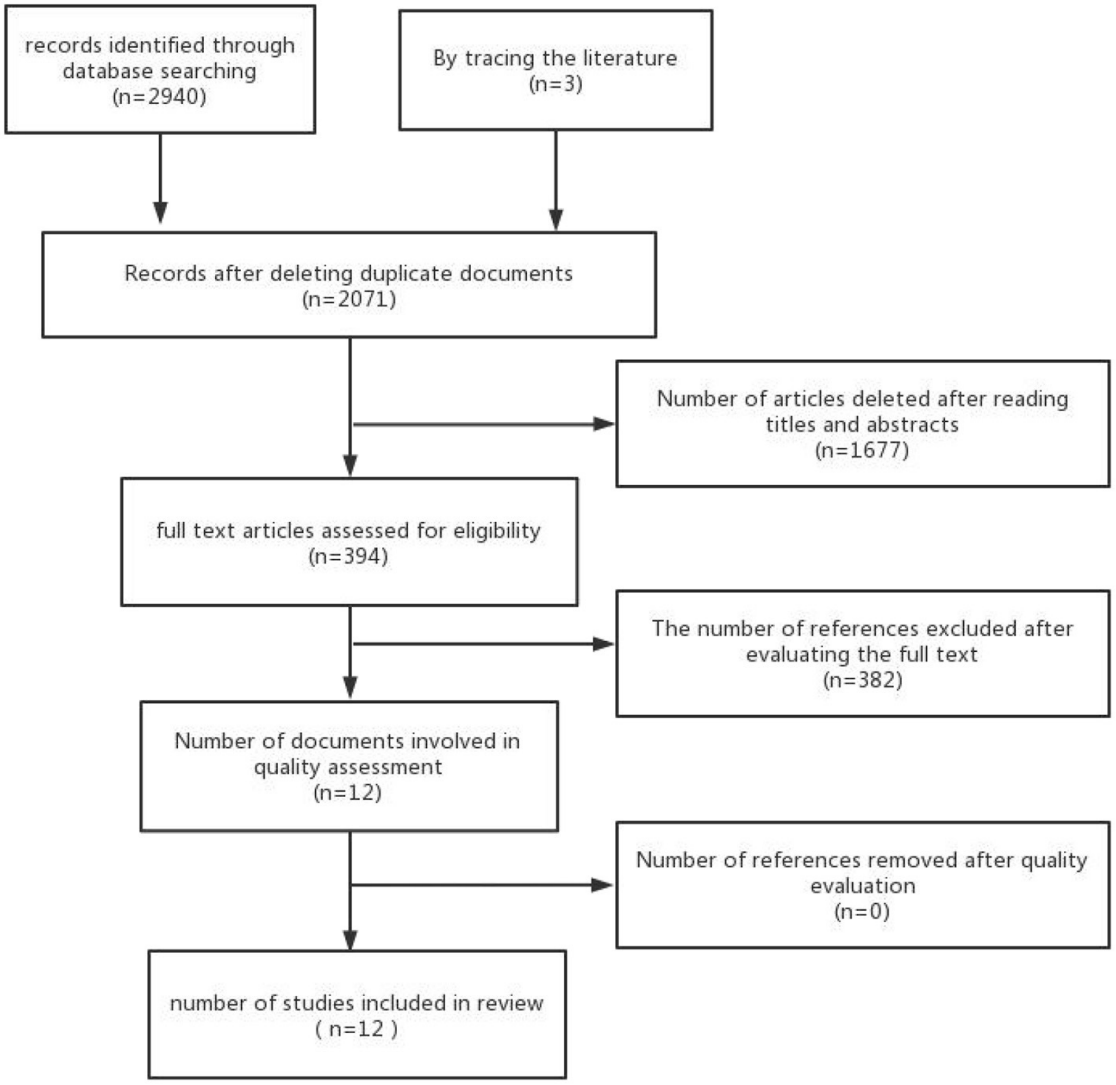
The literature screening steps.

Two researchers screened the retrieved literature according to inclusion and exclusion criteria. In case of disagreement, the third researcher was consulted for judgment. In this search, 2,943 studies were obtained, and 872 duplicated articles were removed. Also, 1,677 irrelevant studies were removed after reading the title and abstract, and 382 inconsistent studies were removed after reading the full text. Finally, 12 studies were obtained after literature quality evaluation. The literature screening steps are shown in Figure 1.

### Quality appraisal

The included studies were independently evaluated by two researchers trained in systematic evidence-based care using the JBI Australian Centre for Evidence-based Health Care Qualitative research Quality evaluation criteria (19). This method evaluates studies from ten aspects, and the results are divided into three aspects: yes, no, and unclear. Each criterion is assigned a corresponding score (yes = 2, no = 0, unclear = 1); the overall score for each study is 20 points. These scores are eventually converted to a percentage system. Finally, a study with a score of 70% was selected and retained. The score was the result of an agreement between two researchers. In case of disagreement, the two researchers further discussed or consulted a third investigator for the verdict. The final evaluation results are shown in Table 1.

**Table 1:** Evaluation of methodological quality.

**Included studies**	**①**	**②**	**③**	**④**	**⑤**	**⑥**	**⑦**	**⑧**	**⑨**	**⑩**	**Results (%)**
Jiang et al. (20)	Y	Y	Y	Y	Y	N	N	Y	U	Y	15/20 (75%)
Xie et al. (21)	Y	Y	Y	Y	Y	N	N	Y	U	Y	15/20 (75%)
Li et al. (22)	Y	Y	Y	Y	Y	N	N	Y	U	Y	15/20 (75%)
Zhang et al. (23)	Y	Y	Y	Y	Y	N	N	Y	U	Y	15/20 (75%)
Xu et al. (24)	Y	Y	Y	Y	Y	N	N	Y	U	Y	15/20 (75%)
Atefi et al. (25)	Y	Y	Y	Y	Y	U	Y	Y	Y	Y	19/20 (95%)
McNeese-Smith (26)	Y	Y	Y	Y	Y	U	U	Y	Y	Y	18/20 (90%)
Fagerberg (27)	Y	Y	Y	Y	Y	U	Y	Y	Y	Y	19/20 (95%)
McKenzie and Addis (28)	Y	Y	Y	Y	Y	U	U	Y	Y	Y	18/20 (90%)
Kristoffersen (29)	Y	Y	Y	Y	Y	U	Y	Y	U	Y	18/20 (90%)
Aagaard et al. (30)	Y	Y	Y	Y	Y	U	Y	Y	Y	Y	19/20 (95%)
Sheng et al. (31)	Y	Y	Y	Y	Y	U	U	Y	Y	Y	18/20 (90%)

JBI Australian Centre for Evidence-based Health Care Qualitative research Quality evaluation criteria (19): ① Is there congruity between the stated philosophical perspective and the research methodology? ② Is there congruity between the research methodology and the research question or objectives? ③ Is there congruity between the research methodology and the methods used to collect data? ④ Is there congruity between the research methodology and the representation and analysis of data? ⑤ Is there congruity between the research methodology and the interpretation of results? ⑥Is there a statement locating the researcher culturally or theoretically? ⑦Is the influence of the researcher on the research, and vice-versa, addressed? ⑧Are participants, and their voices, adequately represented? ⑨Is the research ethical according to current criteria or, for recent studies, and is there evidence of ethical approval by an appropriate body? ⑩Do the conclusions drawn in the research report flow from the analysis, or interpretation, of the data?

### Data extraction

Data extracted in this study included author and year, source, objective, methodology, and conclusion. These results were evaluated by two investigators, and in case of disagreement, by a third investigator. The final results are shown in Table 2.

**Table 2:** Characteristics of the included studies (*n* = 12).

**Reference**	**Origin**	**Aim**	**Methodology**	**Results**
Jiang et al. (20)	China	To explore the sense of professional benefit of nurses in emergency department	Using a phenomenological approach to qualitative research, face-to-face semi-structured in-depth interview. The sample size was 16, including 2 males and 14 females. Age range: 24–47	Four themes emerged: (1) Professional values (2) Organizational support (3) Family gain (4) Good working atmosphere
*Xie* et al. (21)	China	To explore the expectations of operating room nurses for burnout relief	Using a phenomenological approach to qualitative research, face-to-face semi-structured in-depth interview. The sample size was 10, including 1 male and 9 female. Working years 1–25 years	Four themes emerged: (1) Expect support from colleagues (2) Expect support from the head nurse (3) Expect medical coordination and cooperation (3) Expect to improve nurses' professional identity and career planning
Li et al. (22)	China	To explore the occupational benefit of oncology nurses	Using a phenomenological approach to qualitative research, face-to-face semi-structured in-depth interview. The sample size was 15, including 1 male and 14 female. Age range:24–49	Five themes emerged: (1) Specialized nursing knowledge and skills (2) Professional affirmation after helping patients (3) Scientific research ability (4) Teaching ability (5) Peer help and support
Zhang et al. (23)	China	To explore the promoting factors of occupational stability of male nurses in tertiary hospitals	Using a phenomenological approach to qualitative research, face-to-face semi-structured in-depth interview. The sample size was 9, with an age range of 26–31	Seven themes emerged: (1) Nursing makes you valuable (2) Nursing work benefits the family (3) Male nurses have different advantages Satisfactory remuneration package (4) Good working atmosphere (5) Attention and training of leaders (6) Understanding and support from relatives and friends (7) Male nurses are gradually accepted by society
Xu et al. (24)	China	To understand the professional identity of operating room nurses	Using a phenomenological approach to qualitative research, face-to-face semi-structured in-depth interview. The sample size was 9, including 3 males and 6 females. Age range: 23–40	Five themes emerged: (1) The humanistic care I get in my work. (2) In compensation (3) The first aid ability and emergency response-ability (4) Realization of personal value and family gain
Atefi et al. (25)	Malaysia	To explore the job satisfaction of registered nurses in Malaysia	Qualitative research and face-to-face semi-structured in-depth interview were used. The sample size was 46 from operating rooms, intensive care units, and internal medicine.	Three themes emerged: (1) Spiritual feeling (2) Work environment factors (3) Motivation
McNeese-Smith (26)	USA	To explore the job satisfaction and dissatisfaction of nursing staff	Qualitative research and semi-structured taped interviews were used. The sample size was 30, including 28 females and 2 males, with an age range of 31–59. From ICU and internal medicine.	Four themes emerged: (1) Patient care (2) The pace and variety in an acute care environment (3) Relationships with coworkers. (4) Meeting personal and family needs
Fagerberg (27)	Sweden	To explore the work experience and professional identity of nurses	Phenomenological methods of qualitative research were used through annual interviews and student surveys. The sample size was 16. From intensive care units, psychiatric departments, operating rooms, and emergency departments.	Three themes emerged: (1) The meaning of caring for and protecting patients (2) The meaning of work organization in nurses' work (3) The implied meaning of using one's professional role.
McKenzie and Addis (28)	UK	To explore the job satisfaction of inpatient ward nurses in the nephrology department	Using phenomenological methods of qualitative research, face-to-face semi-structured interviews were conducted. The sample size was 12, from the critical ward and general ward of the department of nephrology.	Three themes emerged: (1) Self care (2) Organizational culture (3) Work environment
Kristoffersen (29)	Norway	To explore the influencing factors of nurses' intention to stay at work	The hermeneutic method of qualitative research is adopted. Sample size: 13 people, age range: 26–60, working years: 2–40 years. Mainly from emergency departments, intensive care units, psychiatry, and oncology	Two themes emerged: (1) Acting as a professional contributor (2) Realigning to maintain professional belongingness
Aagaard and Rasmussen (30)	Denmark	To explore the professional identity of anesthesiologists	Using ethnography of qualitative research. The sample size was 12, mainly for breast cancer and gastrointestinal cancer.	Two themes emerged: (1) Gliding between tasks and structures (2) Depending on independence
Sheng et al. (31)	China	To explore the professional identity of Chinese nurses participating in COVID-19 rescue missions	Using the phenomenological method of qualitative research. Through face-to-face and semi-structured interviews, the sample size was 14, all of whom participated in the support work in Wuhan, Hubei province, during the COVID-19 outbreak	Four themes emerged: (1) Impression of exhaustion and fear (2) Feeling the unfairness (3) Perceiving incompetence in the rescue task (4) Unexpected professional benefits

### Data analysis and synthesis

Qualitative research is a broad concept that encompasses a variety of approaches. Researchers argue that qualitative research can provide rich insights and descriptions, but the lack of connections between studies limits its application and progress. Meta-synthesis of qualitative studies can solve this problem to some extent by not only summarizing the results of all qualitative studies but also by interpreting the results of qualitative studies to create new perspectives and so-called “third-level” findings (32). Meta-synthesis is a systematic evaluation method in which the results of two or more studies are classified and reclassified to form a comprehensive result (19). In the first place, the researchers read papers and then re-read them to gain a preliminary understanding. Then, the results of each study are extracted, along with the research results showing that the results of data and text and included in the study of support data consistency between levels by two independent researchers. In case of disagreement, the third researcher was consulted. Each finding was assigned its own level of confidence: both explicitly credible and unsupported (19). Results were then encoded according to their meaning and content. Researchers looked for similarities and differences between the findings and the textual data, and the meanings of the original data set were classified. Next, the categories were read over and over again, searching for similarities and forming comprehensive findings.

## Results

From a total of 2,943 identified studies, 12 were included in the analysis. None of the included studies specified the values and cultural background. In addition, six articles did not indicate whether their studies were approved by ethics bodies. Out of the 12 included studies, 11 were qualitative and one was a mixture of qualitative and quantitative studies. Methodology included descriptive research (*n* = 2), phenomenological research (*n* = 8), ethnography (*n* = 1), and hermeneutic research (n = 1). The studies were from following countries: China (*n* = 6), UK (*n* = 1), Denmark (*n* = 1), Malaysia (*n* = 1), USA (*n* = 1), Sweden (*n* = 1), and Norway (*n* = 1). The study participants ranged in age from 23 to 60 from 1999 to 2021. Sample sizes ranged from 9 to 46. A total of 202 samples were included in the studies. All the included studies focused on nursing staff in critical and critical care departments, and the qualitative research subjects were mainly from operating rooms, intensive care units, emergency departments, oncology departments, nephrology departments, etc., as well as nurses who volunteered during COVID-19 (Table 2). A total of 55 results were extracted from the 12 included literature, and 8 new categories were obtained after synthesizing. Three new themes were obtained after synthesizing 8 new categories: professional identity, social support, and personal growth (Table 3).

**Table 3:** Thematic synthesis findings.

**Descriptive themes**	**Sub-themes**
Professional identity	Proud professional ability
	Increasing professional value
Social support	Friends and family support
	Organizational environmental support
	Peer support
	Support of patients and their families
personal growth	Realize self-worth
	Promote self-development

### Theme 1: Professional identity

#### Proud professional ability

The situation in acute and critical care units is always urgent, and the work environment is complex and stressful. Therefore, it is very important to have good psychological quality, skilled operation technology, and teamwork ability. Having the professional ability recognized and affirmed by people can increase the professional identity of nurses, thus enhancing their sense of professional benefit. “*The most beneficial aspect of my work is the patients' recognition and praise of my work after each operation and education, which makes me more confident and motivated to persist.”* (20); “*I have a lot of knowledge about cancer prevention” and “I have mastered PICC tube placement technology, which is an affirmation of my professional ability”* (22). Many nurses express that they have a certain degree of autonomy in the clinical decision-making process and can use their knowledge and experience to save patients' lives in emergency situations when a life-threatening situation occurs and the doctor has not yet arrived (30). This independence greatly enhances the sense of accomplishment and career benefit and improves the quality of nursing.

#### Increasing professional value

Nurses in acute and critical care departments usually deal with critically ill patients, so after treatment and nursing, patients' rehabilitation and discharge may increase their professional sense of achievement, which is also a reflection of their professional value. “*He was a very critical patient, but he got better through our daily care, was transferred out of ICU and discharged. I feel a sense of accomplishment every time I see him”* (23). Many nurses said their professional value was reflected in saving lives (20). In addition, nurses said they felt their profession was valuable because their knowledge and clinical experience could help those around them, reducing the need for hospital visits (20) “*My relative's enterostomy was performed under my guidance, and there were no complications* (22).”

### Theme 2: Social support

#### Friends and family support

Family and friends are important pillars of one's social support. In fact, the support of relatives and friends can provide a lot of comfort and motivation for nurses, thus strengthening their willingness to stay and enhancing the sense of professional benefit. “*My wife is a nurse. She understands me and supports me because she is in the same profession”* (23); and “*The elderly in my family respect medical care and let me do my job well. My family supports me. That's enough* (21).”

#### Organizational environmental support

Organizational support is also an important factor affecting the sense of career benefit. If the leaders and management of a hospital attach importance to the development and needs of employees, they will achieve certain accomplishments through teamwork as employees feel cared for (23). “*Many new hires in nephrology can feel a lot of stress when they first come in, but the education team guides you through the work, helping you to familiarize yourself with the problems you'll encounter on the job* (28).” In addition, hospital management's bonus setting and medical equipment upgrades can undoubtedly improve nurses' sense of professional benefit. “*I'm in the emergency department, benefiting from all kinds of subsidies; the salary level is basically satisfactory* *(**23**)*.” In acute and critical care units, patients often need to be rescued at any time or need high-end equipment for auxiliary diagnosis and treatment, so rich medical resources are indispensable. “*We have a good nursing facility and all the equipment we need to provide safe patient care* (28).”

#### Peer support

Peer support is often helpful for emotional and stress support at work, and is also one of the conditions for maintaining a good working atmosphere and high work efficiency. Understanding each other and sharing life experiences among colleagues can greatly relieve occupational stress: “*I am a nurse from other places. Nurses in our department work side by side and tend to cooperate. The oncology department has a heavy workload, but we still manage to work closely. I am not from around here, but I still feel accepted* (20).” Similarly, the support of the head nurse is also of great help in improving the sense of professional benefit among nurses: “*This was my fourth year at work, and the head nurse assigned the task of instrument management to me. I felt appreciated, which encouraged me to try to do better* *(**23**)*.” In addition, nurses say that approval from doctors can also motivate them (24).

#### Support from patients and their families

Mutual understanding and respect between nurses and patients, and mutual help and understanding between family members and nurses can make nurses feel affirmed and supported, which further enhances their professional benefit (26). “*I found meaning and confidence in my work with the active participation of my patients in the health promotion* (31).” Nurse-patient contradiction is an important problem in today's society. The increasingly acute nurse-patient contradiction directly affects the turnover rate of nurses. Therefore, a good nurse-patient relationship is essential for both individuals and organizations.

### Theme 3: Personal growth

#### Promote self development

The professional ability and psychological quality accumulated by nurses in acute and critical care help them improve their professional identity to a certain extent. For example: “*I think my reflexes are faster. That's what my major gives me”* (20); other nurses reported that their calm response and treatment in the first aid processes, as well as their decisive decision-making power and strong psychological abilities instilled a sincere sense of professional pride in them. Some nurses with rich theoretical knowledge and innovative spirit took the initiative to participate in scientific research activities, paper writing, and teaching, finding new professional significance and satisfaction in the process of learning and studying, which also strengthens their professional belongingness. “I now have students of my own, and I hear they want to be like me in the future, which is comforting (26).” Some hospitals provide nurses with various opportunities for further studying and learning, to a certain extent, thus stimulating the professional confidence of nurses and their personal growth (22).

#### Realization of self-worth

Engaging in nursing work helps nurses to find their sense of value and mission to a certain extent, thus enhancing their sense of professional benefit. “*Because our department often needs consultation, I also get to know many doctors, which is convenient to provide medical consultation for people around me, making me feel that my work is very valuable* (21).” Many nurses say they choose to nurse and now they are stuck with it because it is stable and pays well (24). As health workers, their relatives and families can also reap the benefits. “*Sometimes friends and relatives ask me about cancer, and I can try to help them”* (22). Nurses who participated in the rescue during the epidemic believe that unexpected rewards, whether in a material form or spiritual feedback, made them realize their own value (31). In addition, due to the physical advantages of male nurses, they reported a greatly improved sense of professional benefits (23). Other nurses found nursing to be a good fit for their personality and religious beliefs. Some said they had grown up empathizing with others, eager to understand and help them, and that nursing allowed them to fulfill their dreams (27). Other nurses said they were not nursing for money or material things but for firmer beliefs, such as invigorating energy and serving God (26).

## Discussion

The purpose of this study was to elucidate the problems related to occupational benefit perception of acute and critical care nurses so as to find a more suitable management method for the hospital nursing team. Moreover, these data provide more comprehensive information for hospital human resource management, which in turn could promote the improvement of medical level and the development of high-quality nursing career.

Due to the special environment of acute and critical care, nurses in the department must have higher specialized skills, theoretical knowledge, and rich clinical experience. Meanwhile, they are expected to keep up with current events, understand the cutting-edge knowledge of the development of acute and critical care medicine, and timely update themselves on various emergency guidelines. Previous studies have suggested that the specialized skills of nurses and the value they show in the process of work can greatly improve their sense of professional benefit (20). Therefore, nursing managers should provide more opportunities for nurses to learn and further their studies so as to meet their needs for self-improvement. At the same time, managers should pay attention to stress and negative emotions and successful ways to avoid or deal with them. They can try to guide nurses to correctly view pressure and deal with it. In addition, managers can also provide more psychological decompression measures, such as music therapy, meditation, group counseling, etc., so as to prevent job burnout caused by long-term pressure load and improve nurses' resilience. Nursing managers can also organize lectures related to professional interests, enhance the importance of role models, use workshops or online meetings to publicize the outstanding deeds of outstanding nursing workers, and help nurses to find a sense of honor and holiness of the profession, thus enhancing their professional identity and improving the quality of nursing.

Hospital managers should also pay more attention to the psychological needs of nurses in their future work, which might be smaller and related to salary and treatment, the satisfaction of a harmonious working atmosphere with peer support, or larger such as striving for more independent decision-making opportunities, in order to allow nurses to combine knowledge with practice to a greater extent so as to achieve their own life value. All of these have a significant influence on nurses' sense of professional benefit. If we take Maslow's hierarchy of needs (33) as an example, we can see that it has a lot of links with the sense of benefit of the nursing profession. The first thing nurses need is their physiological needs to be met, followed by a steady income that can ensure the availability of food, clothing, housing, and transportation, thus improving their quality of life. Second, they have safety needs, i.e., nurses and relatives, and friends can experience a certain degree of worry about their health and medical treatment. Next, there is the need for love and belonging; nurses can feel the sense of respect brought on by this job to a certain extent when they receive the attention and affirmation from head nurses and managers, as well as the understanding and support or even admiration from family members and patients. Management by Wandering Around (MB-WA) is an effective form of hospital comprehensive management tool used by nursing managers. The figures of this tool often appear in the field of vision of nurses, greatly increasing their work confidence and security. Also, in this process, which includes listening and guidance, the work enthusiasm of nurses is greatly improved, including an increased professional sense of belonging among nurses (34). The support of the external environment is also a part of the attribution needs of nurses. Only when managers attach importance to the development of hospitals and nursing undertakings, actively prepare funds for this, and purchase advanced equipment can nurses feel cared for and supported. Nurses also need respect, where nurses establish spiritual links with their peers in daily work. They do not only coordinate and cooperate in terms of work but also become friends with each other. Finally, there is the top-level need, which namely includes the need for self-actualization, i.e., the realization of professional honor and self-worth mentioned above. Hospital managers should take corresponding measures to meet the needs of these levels in different degrees to improve the career satisfaction of nurses, thus reducing the dismission rate and attracting more and more excellent nursing talents to join the team.

In addition to paying attention to the psychological needs of nurses, hospital managers should also actively build a good working atmosphere for staff and recruit more excellent nursing talents to enrich and develop the nursing team. It is necessary to strengthen the guidance of public opinion, improve the promotion and personnel selection system, and make it fair, open, and just so that talented and competent people can give fully showcase their professional advantages (35). Since the shortage of nursing staff often leads to work overload and occupational pressure (21), which is also the main reason for job burnout, managers should start from multiple aspects, adjusting the ratio of nurse to patient, reducing the workload of nurses as much as possible and control it within a reasonable range. As acute critical care nurses work in a special working environment (36), with relatively large occupational pressure and complex patient situation, it is necessary to find ways to improve the work system and their job satisfaction. In today's society, the nurse-patient contradiction is more prominent (37). Managers should strengthen the guidance of public opinion, promote the deeds of outstanding nursing workers, guide nurses to take this as an example, guide senior nurses to share their experience with new nurses, and provide as many opportunities as possible for nurses to learn nurse-patient communication skills and call for the establishment of a harmonious relationship. Although autonomous decision-making is rarely mentioned in Asian cultures as part of nurses' work (25), studies in western countries mention the influence of independent clinical decision-making on nurses' sense of professional benefit and professional pride. As the first country that develop advanced practice nurses, the United States has developed a sound qualification certification system, which can be used for reference by nursing managers in other countries. Through professional training, nurses can improve their educational background by gaining expert knowledge and skills to make better clinical decisions (38). Managers can also do their best to provide opportunities for nurses and encourage them to further study, carry out research projects and solo teaching tasks to develop more nursing talents (39), and promote professional pride.

Because of the influence of the COVID-19 pandemic, nursing staff should also pay attention to protect themselves in the process of working so as not to get infected. Managers need to attach importance to the nurses' needs, provide sufficient protection material, arrange more lectures on epidemic prevention and control, and provide nurses with timely guidance on how to deal with negative emotions and pressure.

As some studies pointed out that the professional development of nurses and the realization of self-worth may also depend on childhood education related to religion, inner personality, and growth environment, managers might also pay attention to nursing workers' intrinsic personalities, and religious beliefs (27) when providing them with learning opportunities and assignments. They should also teach nurses according to their aptitude whenever possible so as to let everybody fully display their talents and better ensure team coordination. As with the progress of modern medical concepts, more and more male nurses are pursuing nursing careers. Therefore, managers should recognize their gender strengths and encourage them to continue to excel in their positions.

In a word, nursing managers should focus on the acute and critical care nurses' career benefit. They should take a series of practical and effective measures for management and reform, which will improve the nurse's resilience, increase the career identity, and encourage nurses to put more energy and attention into work, thus improving the quality of care and patients' satisfaction.

## Limitations

The purpose of this study is to explore the professional benefit of nurses in acute and critical care. A total of 12 studies from 7 countries were included in the analysis. Due to language restrictions, non-Chinese and non-English literature was excluded. Despite our efforts to search for as comprehensive literature as possible, there is no doubt that some relevant documents were missed.

## Conclusion

This is a synthesis of qualitative studies on the perception of professional benefits among acute and critical care nurses. We identified three new themes: professional identity, social support, and personal growth. Hospital managers should pay attention to the positive emotional experience of nurses in work and based on this, provide practical and beneficial protection for nurses from the aspects of salary, learning opportunities, working environment, social support and internal personality, stimulate work enthusiasm, guide nurses to correctly face negative emotions and occupational pressure, and improve the sense of professional benefit. In future research, we will continue to explore the long-term positive psychological experience of acute and intensive care specialist nurses. These results can be used as a guide for nursing managers to develop more perfect management plans. When the positive experience of nurses is strengthened, the professional expectation is met, and the sense of professional benefit is improved, the turnover rate of nurses can be effectively reduced, the professional identity of nurses can be improved, so as to be more engaged in clinical work, improve the clinical outcome of patients, and promote the progress of medical level and the high-quality development of nursing career.

## Data availability statement

The original contributions presented in the study are included in the article/supplementary material, further inquiries can be directed to the corresponding authors.

## Author contributions

SL: conceptualization, methodology, formal analysis, writing—original draft, and writing—review and editing. XD: conceptualization, methodology, writing—original draft, and writing—review and editing. PH and HS: conceptualization, methodology, formal analysis, and writing—review and editing. JJ and LZ: methodology, formal analysis, and writing—review and editing. All authors contributed to the article and approved the submitted version.

## Conflict of interest

The authors declare that the research was conducted in the absence of any commercial or financial relationships that could be construed as a potential conflict of interest.

## Publisher's note

All claims expressed in this article are solely those of the authors and do not necessarily represent those of their affiliated organizations, or those of the publisher, the editors and the reviewers. Any product that may be evaluated in this article, or claim that may be made by its manufacturer, is not guaranteed or endorsed by the publisher.
